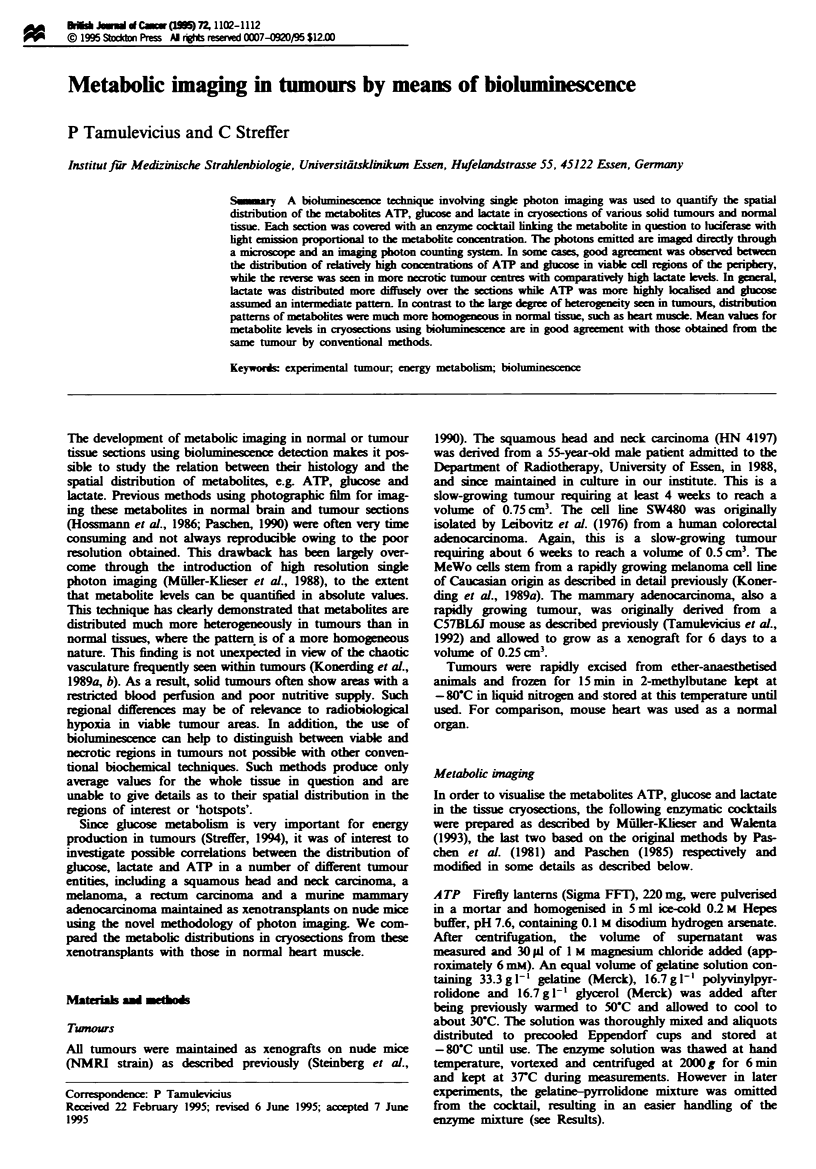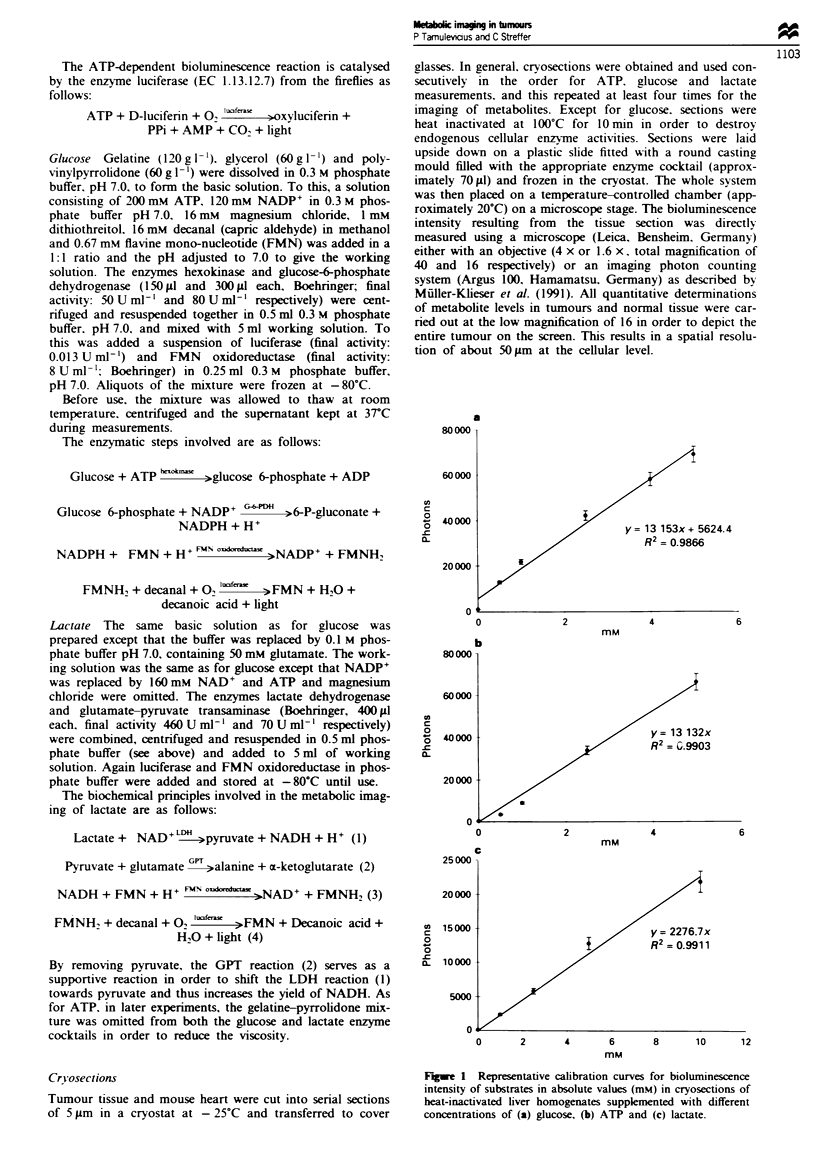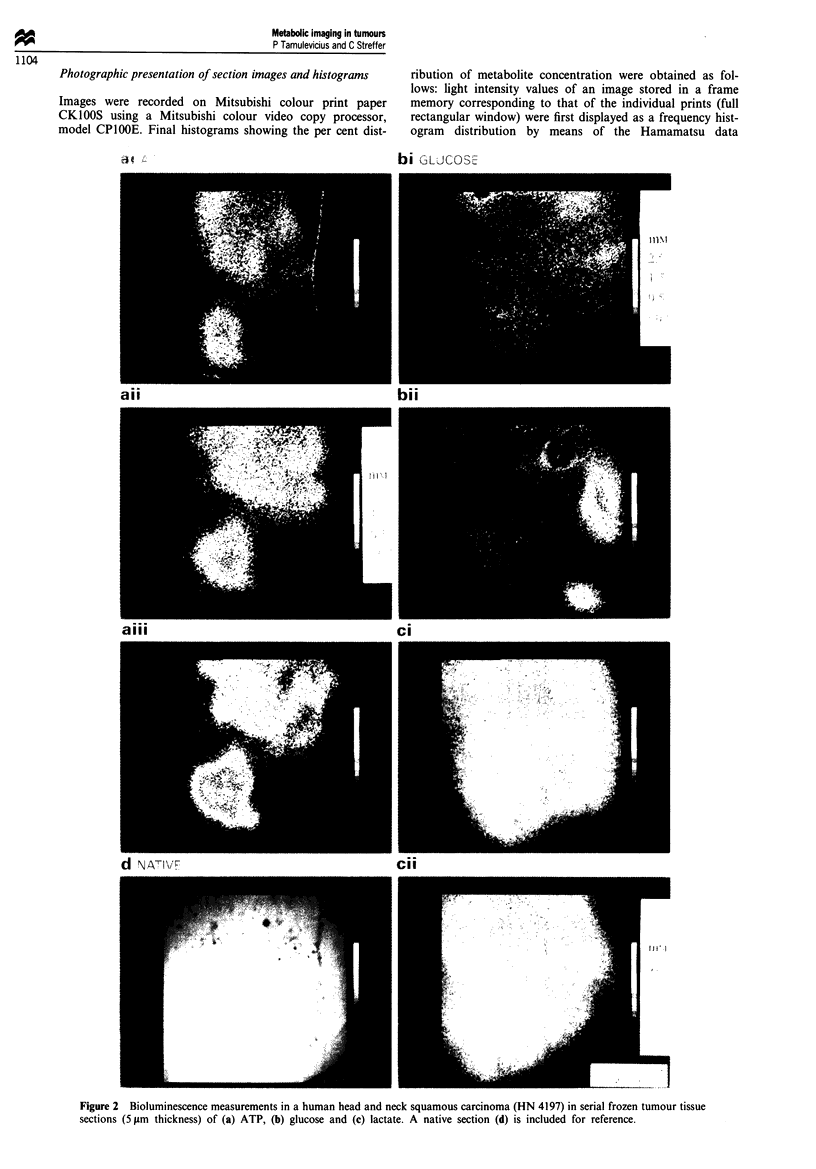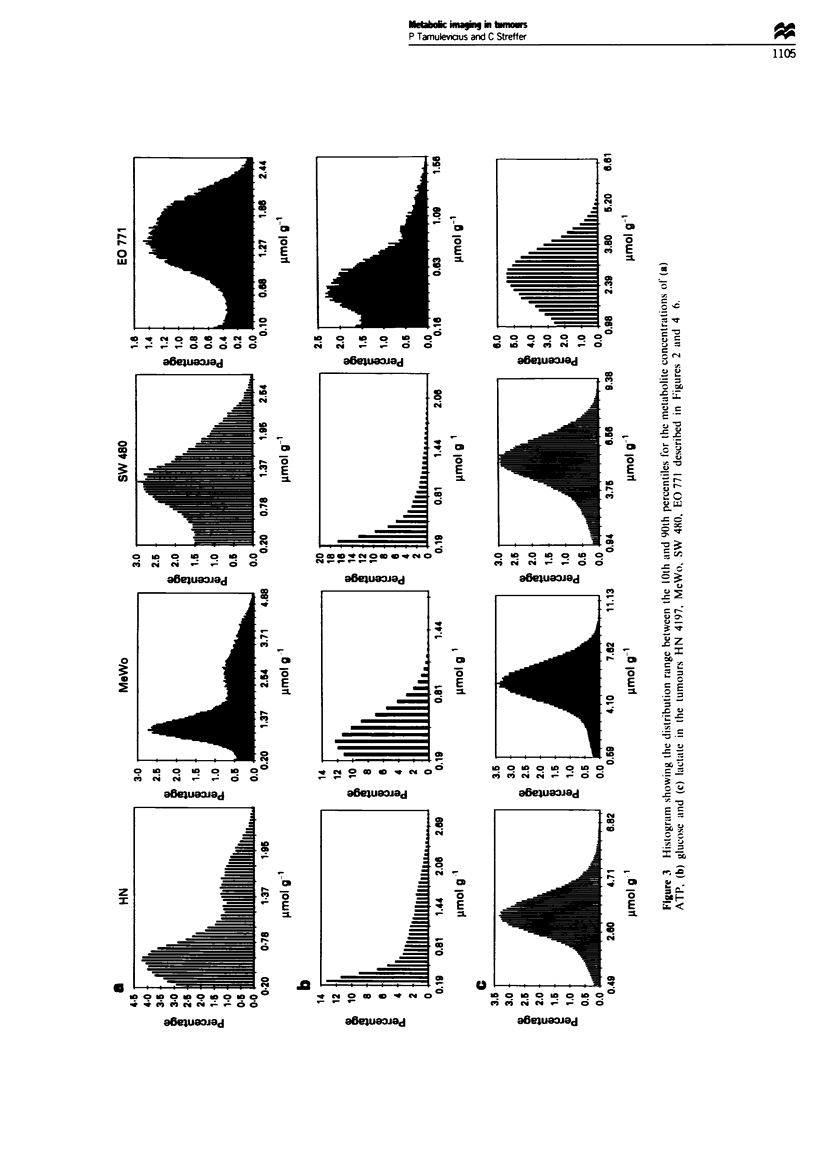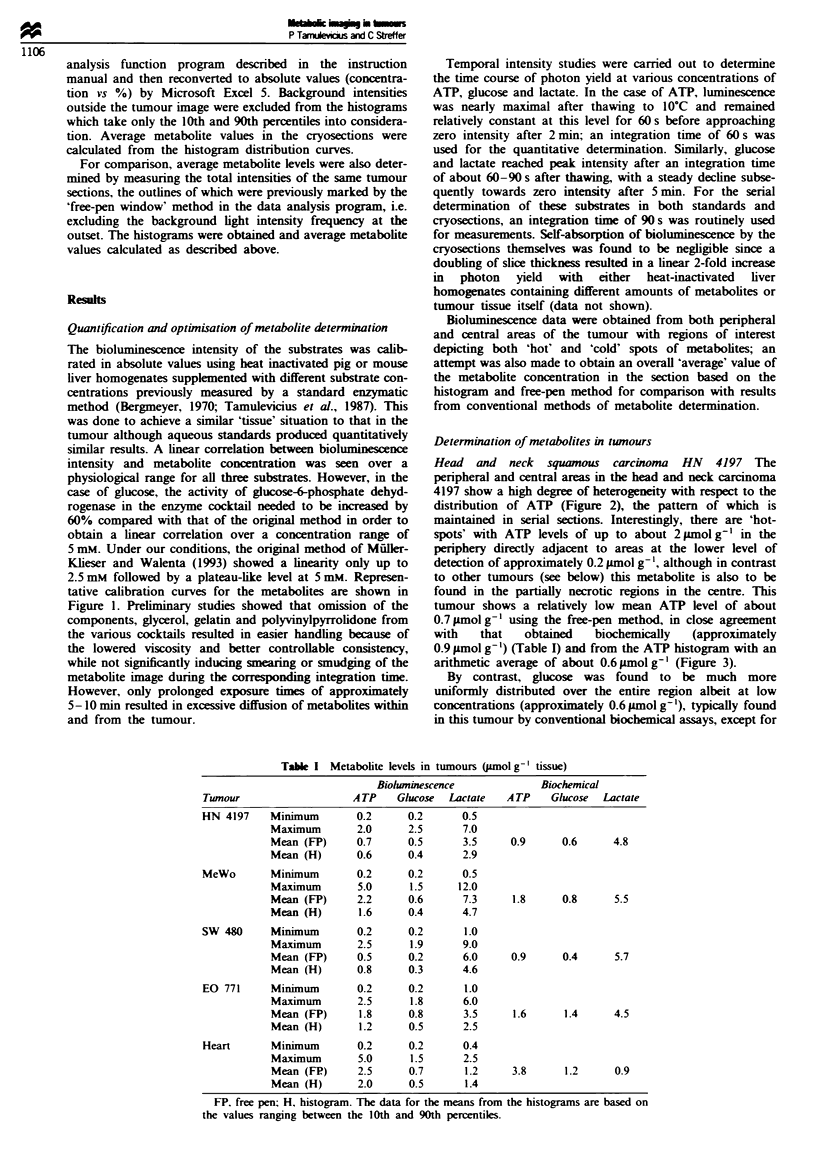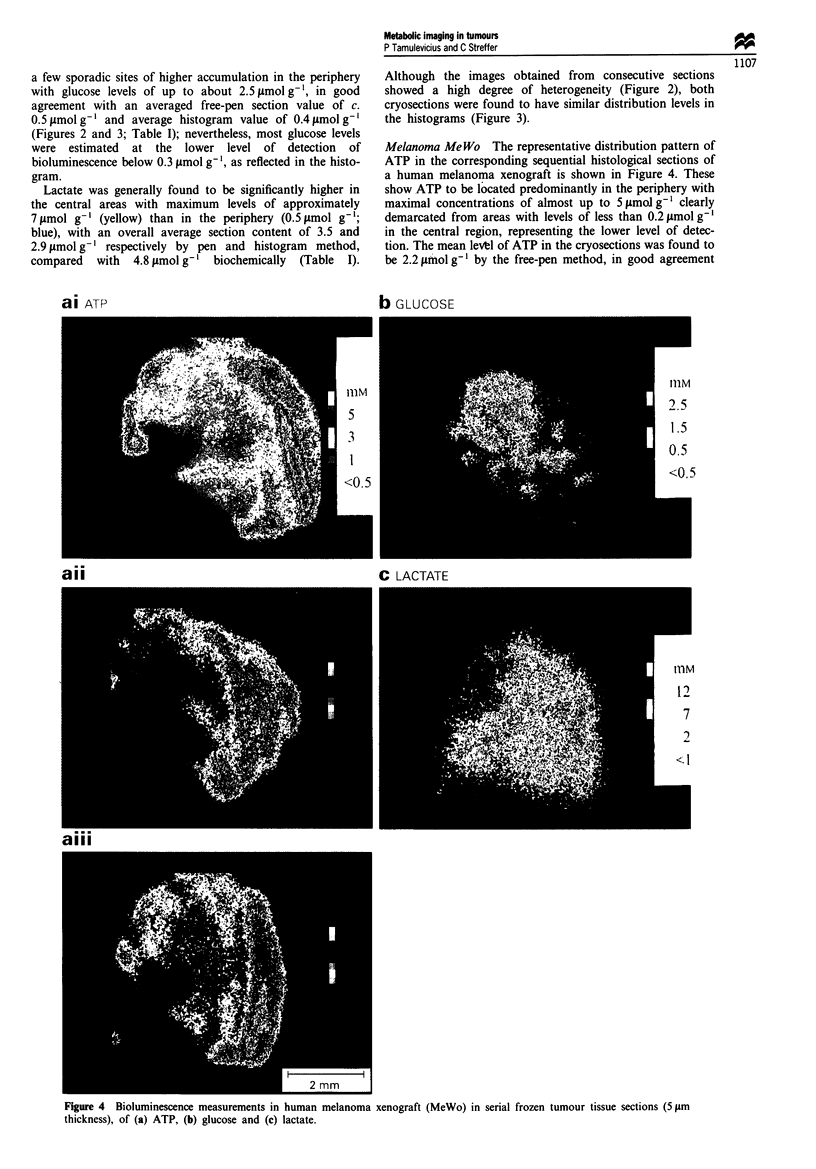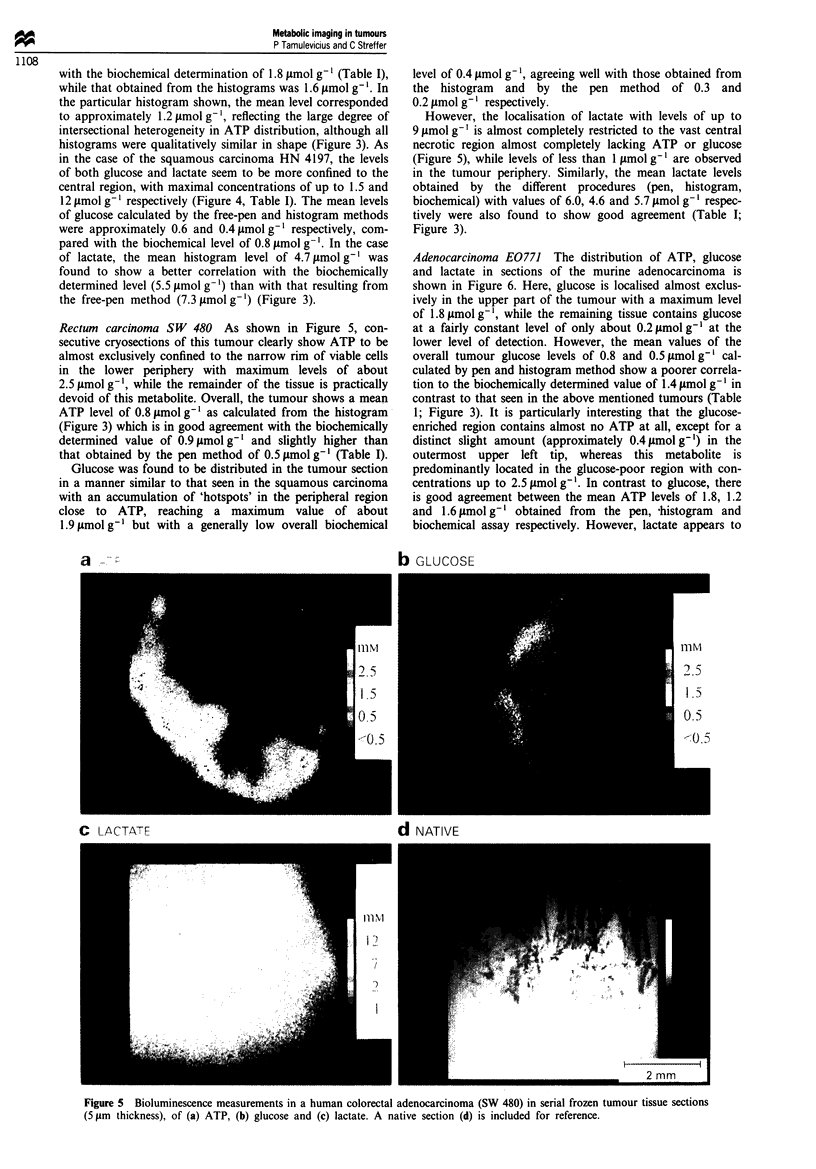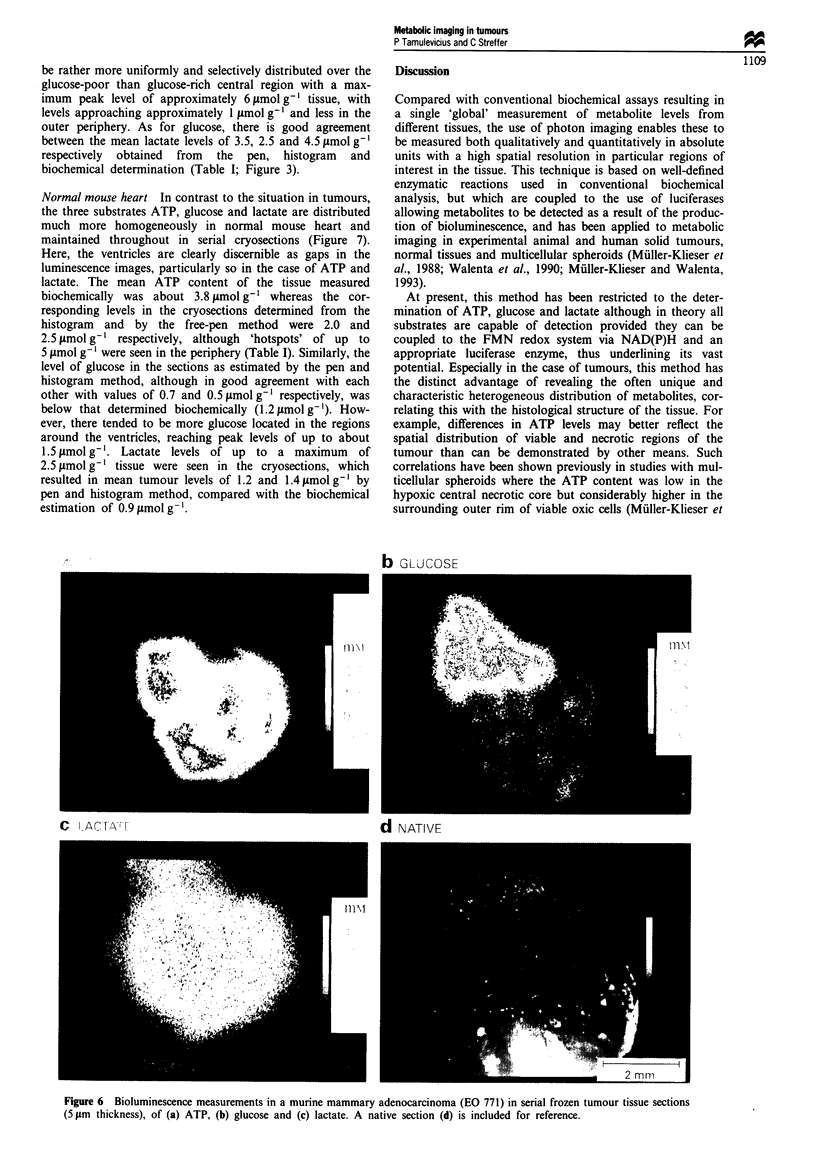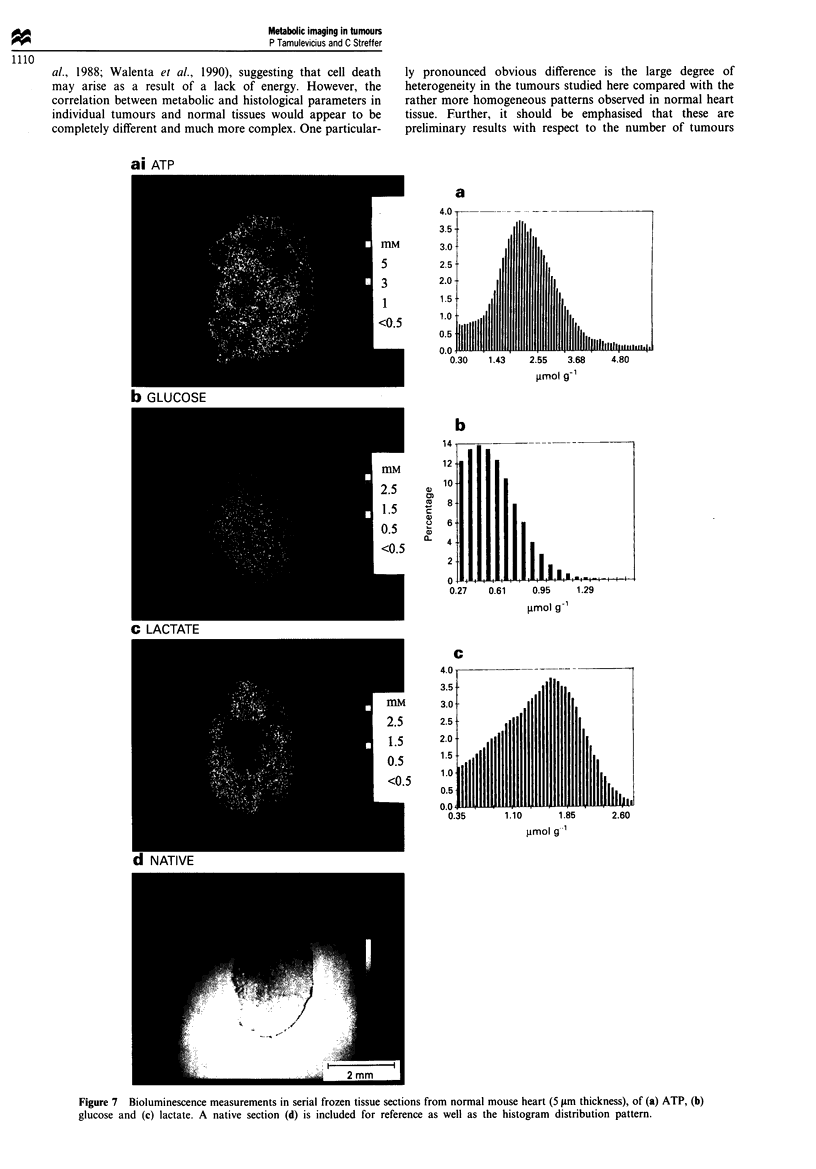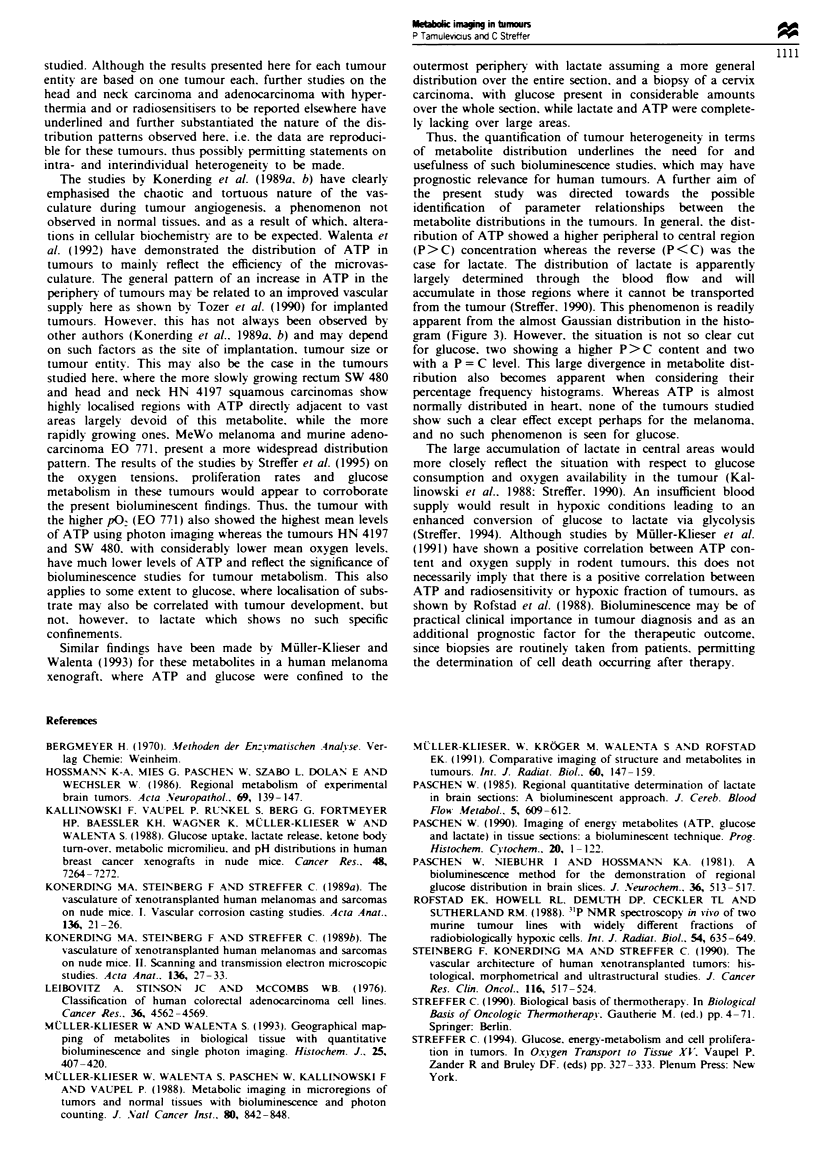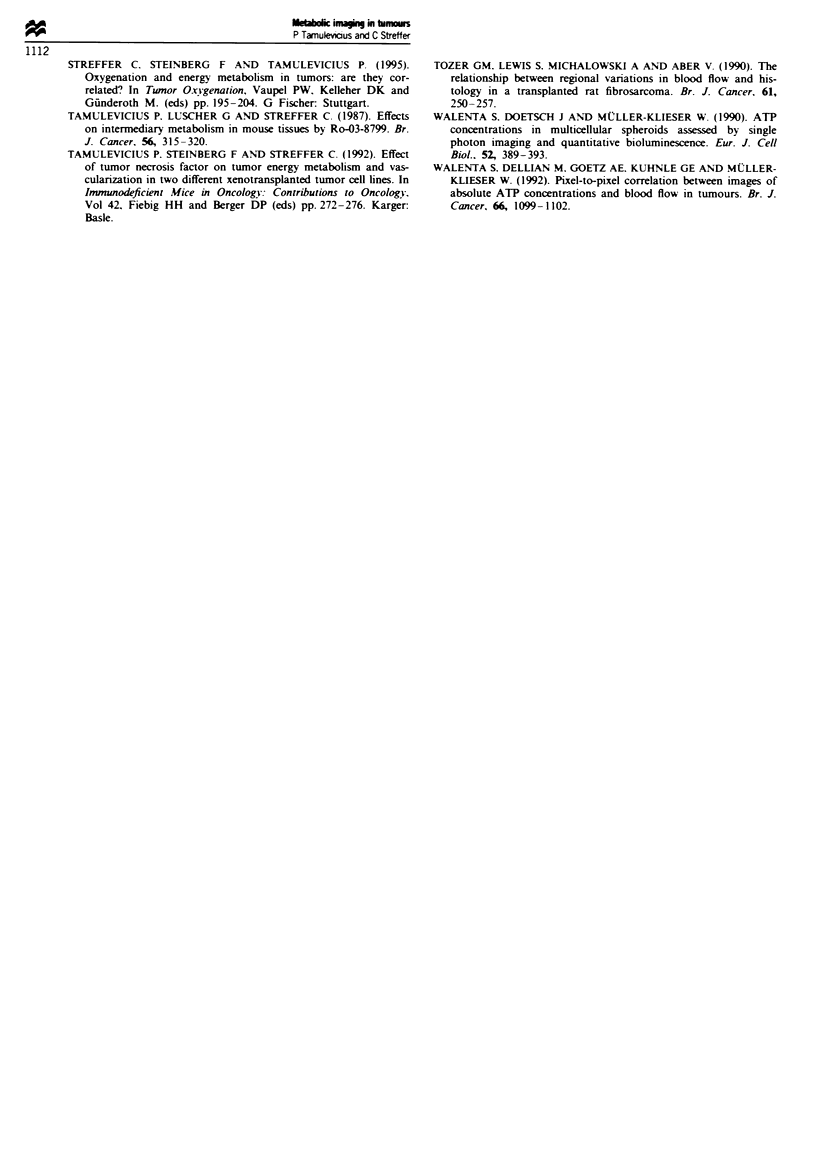# Metabolic imaging in tumours by means of bioluminescence.

**DOI:** 10.1038/bjc.1995.472

**Published:** 1995-11

**Authors:** P. Tamulevicius, C. Streffer

**Affiliations:** Institut für Medizinische Strahlenbiologie, Universitätsklinikum Essen, Germany.

## Abstract

**Images:**


© 1995 Stockton Press All rights reserved 0007–0920/95 $12.00

### Metabolic imaging in tumours by means of bioluminescence

P Tamulevicius and C Streffer

*Institut für Medizinische Strahlenbiologie, Universitätsklinikum Essen, Hufelandstrasse 55, 45122 Essen, Germany*

**Summary** A bioluminescence technique involving single photon imaging was used to quantify the spatial distribution of the metabolites ATP, glucose and lactate in cryosections of various solid tumours and normal tissue. Each section was covered with an enzyme cocktail linking the metabolite in question to luciferase with light emission proportional to the metabolite concentration. The photons emitted are imaged directly through a microscope and an imaging photon counting system. In some cases, good agreement was observed between the distribution of relatively high concentrations of ATP and glucose in viable cell regions of the periphery, while the reverse was seen in more necrotic tumour centres with comparatively high lactate levels. In general, lactate was distributed more diffusely over the sections while ATP was more highly localised and glucose assumed an intermediate pattern. In contrast to the large degree of heterogeneity seen in tumours, distribution patterns of metabolites were much more homogeneous in normal tissue, such as heart muscle. Mean values for metabolite levels in cryosections using bioluminescence are in good agreement with those obtained from the same tumour by conventional methods.

**Keywords:** experimental tumour; energy metabolism; bioluminescence

The development of metabolic imaging in normal or tumour tissue sections using bioluminescence detection makes it possible to study the relation between their histology and the spatial distribution of metabolites, e.g. ATP, glucose and lactate. Previous methods using photographic film for imaging these metabolites in normal brain and tumour sections (Hossmann *et al.*, 1986; Paschen, 1990) were often very time consuming and not always reproducible owing to the poor resolution obtained. This drawback has been largely overcome through the introduction of high resolution single photon imaging (Müller-Klieser *et al.*, 1988), to the extent that metabolite levels can be quantified in absolute values. This technique has clearly demonstrated that metabolites are distributed much more heterogeneously in tumours than in normal tissues, where the pattern is of a more homogeneous nature. This finding is not unexpected in view of the chaotic vasculature frequently seen within tumours (Konerding *et al.*, 1989*a*, *b*). As a result, solid tumours often show areas with a restricted blood perfusion and poor nutritive supply. Such regional differences may be of relevance to radiobiological hypoxia in viable tumour areas. In addition, the use of bioluminescence can help to distinguish between viable and necrotic regions in tumours not possible with other conventional biochemical techniques. Such methods produce only average values for the whole tissue in question and are unable to give details as to their spatial distribution in the regions of interest or 'hotspots'.

Since glucose metabolism is very important for energy production in tumours (Streffer, 1994), it was of interest to investigate possible correlations between the distribution of glucose, lactate and ATP in a number of different tumour entities, including a squamous head and neck carcinoma, a melanoma, a rectum carcinoma and a murine mammary adenocarcinoma maintained as xenotransplants on nude mice using the novel methodology of photon imaging. We compared the metabolic distributions in cryosections from these xenotransplants with those in normal heart muscle.

### Materials and methods

### *Tumours*

All tumours were maintained as xenografts on nude mice (NMRI strain) as described previously (Steinberg *et al.*, 1990). The squamous head and neck carcinoma (HN 4197) was derived from a 55-year-old male patient admitted to the Department of Radiotherapy, University of Essen, in 1988, and since maintained in culture in our institute. This is a slow-growing tumour requiring at least 4 weeks to reach a volume of 0.75 $cm^3$. The cell line SW480 was originally isolated by Leibovitz *et al.* (1976) from a human colorectal adenocarcinoma. Again, this is a slow-growing tumour requiring about 6 weeks to reach a volume of 0.5 $cm^3$. The MeWo cells stem from a rapidly growing melanoma cell line of Caucasian origin as described in detail previously (Konerding *et al.*, 1989*a*). The mammary adenocarcinoma, also a rapidly growing tumour, was originally derived from a C57BL6J mouse as described previously (Tamulevicius *et al.*, 1992) and allowed to grow as a xenograft for 6 days to a volume of 0.25 $cm^3$.

Tumours were rapidly excised from ether-anaesthetised animals and frozen for 15 min in 2-methylbutane kept at −80°C in liquid nitrogen and stored at this temperature until used. For comparison, mouse heart was used as a normal organ.

### *Metabolic imaging*

In order to visualise the metabolites ATP, glucose and lactate in the tissue cryosections, the following enzymatic cocktails were prepared as described by Müller-Klieser and Walenta (1993), the last two based on the original methods by Paschen *et al.* (1981) and Paschen (1985) respectively and modified in some details as described below.

*ATP* Firefly lanterns (Sigma FFT), 220 mg, were pulverised in a mortar and homogenised in 5 ml ice-cold 0.2 M Hepes buffer, pH 7.6, containing 0.1 M disodium hydrogen arsenate. After centrifugation, the volume of supernatant was measured and 30 μl of 1 M magnesium chloride added (approximately 6 mM). An equal volume of gelatine solution containing 33.3 g $l^{-1}$ gelatine (Merck), 16.7 g $l^{-1}$ polyvinylpyrrolidone and 16.7 g $l^{-1}$ glycerol (Merck) was added after being previously warmed to 50°C and allowed to cool to about 30°C. The solution was thoroughly mixed and aliquots distributed to precooled Eppendorf cups and stored at −80°C until use. The enzyme solution was thawed at hand temperature, vortexed and centrifuged at 2000 *g* for 6 min and kept at 37°C during measurements. However in later experiments, the gelatine–pyrrolidone mixture was omitted from the cocktail, resulting in an easier handling of the enzyme mixture (see Results).

Correspondence: P Tamulevicius
Received 22 February 1995; revised 6 June 1995; accepted 7 June 1995

The ATP-dependent bioluminescence reaction is catalysed by the enzyme luciferase (EC 1.13.12.7) from the fireflies as follows:

$$\text{ATP} + \text{D-luciferin} + O_2 \xrightarrow{\text{luciferase}} \text{oxyluciferin} + \text{PPi} + \text{AMP} + CO_2 + \text{light}$$

*Glucose* Gelatine ($120\ g\ l^{-1}$), glycerol ($60\ g\ l^{-1}$) and polyvinylpyrrolidone ($60\ g\ l^{-1}$) were dissolved in 0.3 M phosphate buffer, pH 7.0, to form the basic solution. To this, a solution consisting of 200 mM ATP, 120 mM $NADP^+$ in 0.3 M phosphate buffer pH 7.0, 16 mM magnesium chloride, 1 mM dithiothreitol, 16 mM decanal (capric aldehyde) in methanol and 0.67 mM flavine mono-nucleotide (FMN) was added in a 1:1 ratio and the pH adjusted to 7.0 to give the working solution. The enzymes hexokinase and glucose-6-phosphate dehydrogenase (150 µl and 300 µl each, Boehringer; final activity: $50\ U\ ml^{-1}$ and $80\ U\ ml^{-1}$ respectively) were centrifuged and resuspended together in 0.5 ml 0.3 M phosphate buffer, pH 7.0, and mixed with 5 ml working solution. To this was added a suspension of luciferase (final activity: $0.013\ U\ ml^{-1}$) and FMN oxidoreductase (final activity: $8\ U\ ml^{-1}$; Boehringer) in 0.25 ml 0.3 M phosphate buffer, pH 7.0. Aliquots of the mixture were frozen at −80°C.

Before use, the mixture was allowed to thaw at room temperature, centrifuged and the supernatant kept at 37°C during measurements.

The enzymatic steps involved are as follows:

$$\text{Glucose} + \text{ATP} \xrightarrow{\text{hexokinase}} \text{glucose 6-phosphate} + \text{ADP}$$

$$\text{Glucose 6-phosphate} + NADP^+ \xrightarrow{\text{G-6-PDH}} \text{6-P-gluconate} + NADPH + H^+$$

$$NADPH + FMN + H^+ \xrightarrow{\text{FMN oxidoreductase}} NADP^+ + FMNH_2$$

$$FMNH_2 + \text{decanal} + O_2 \xrightarrow{\text{luciferase}} FMN + H_2O + \text{decanoic acid} + \text{light}$$

*Lactate* The same basic solution as for glucose was prepared except that the buffer was replaced by 0.1 M phosphate buffer pH 7.0, containing 50 mM glutamate. The working solution was the same as for glucose except that $NADP^+$ was replaced by 160 mM $NAD^+$ and ATP and magnesium chloride were omitted. The enzymes lactate dehydrogenase and glutamate–pyruvate transaminase (Boehringer, 400 µl each, final activity $460\ U\ ml^{-1}$ and $70\ U\ ml^{-1}$ respectively) were combined, centrifuged and resuspended in 0.5 ml phosphate buffer (see above) and added to 5 ml of working solution. Again luciferase and FMN oxidoreductase in phosphate buffer were added and stored at −80°C until use.

The biochemical principles involved in the metabolic imaging of lactate are as follows:

$$\text{Lactate} + NAD^+ \xrightarrow{\text{LDH}} \text{pyruvate} + NADH + H^+ \quad (1)$$

$$\text{Pyruvate} + \text{glutamate} \xrightarrow{\text{GPT}} \text{alanine} + \alpha\text{-ketoglutarate} \quad (2)$$

$$NADH + FMN + H^+ \xrightarrow{\text{FMN oxidoreductase}} NAD^+ + FMNH_2 \quad (3)$$

$$FMNH_2 + \text{decanal} + O_2 \xrightarrow{\text{luciferase}} FMN + \text{Decanoic acid} + H_2O + \text{light} \quad (4)$$

By removing pyruvate, the GPT reaction (2) serves as a supportive reaction in order to shift the LDH reaction (1) towards pyruvate and thus increases the yield of NADH. As for ATP, in later experiments, the gelatine–pyrrolidone mixture was omitted from both the glucose and lactate enzyme cocktails in order to reduce the viscosity.

### *Cryosections*

Tumour tissue and mouse heart were cut into serial sections of 5 µm in a cryostat at −25°C and transferred to cover glasses. In general, cryosections were obtained and used consecutively in the order for ATP, glucose and lactate measurements, and this repeated at least four times for the imaging of metabolites. Except for glucose, sections were heat inactivated at 100°C for 10 min in order to destroy endogenous cellular enzyme activities. Sections were laid upside down on a plastic slide fitted with a round casting mould filled with the appropriate enzyme cocktail (approximately 70 µl) and frozen in the cryostat. The whole system was then placed on a temperature-controlled chamber (approximately 20°C) on a microscope stage. The bioluminescence intensity resulting from the tissue section was directly measured using a microscope (Leica, Bensheim, Germany) either with an objective (4 × or 1.6 ×, total magnification of 40 and 16 respectively) or an imaging photon counting system (Argus 100, Hamamatsu, Germany) as described by Müller-Klieser *et al.* (1991). All quantitative determinations of metabolite levels in tumours and normal tissue were carried out at the low magnification of 16 in order to depict the entire tumour on the screen. This results in a spatial resolution of about 50 µm at the cellular level.

**Figure 1** Representative calibration curves for bioluminescence intensity of substrates in absolute values (mM) in cryosections of heat-inactivated liver homogenates supplemented with different concentrations of (**a**) glucose, (**b**) ATP and (**c**) lactate.

*Photographic presentation of section images and histograms*

Images were recorded on Mitsubishi colour print paper CK100S using a Mitsubishi colour video copy processor, model CP100E. Final histograms showing the per cent distribution of metabolite concentration were obtained as follows: light intensity values of an image stored in a frame memory corresponding to that of the individual prints (full rectangular window) were first displayed as a frequency histogram distribution by means of the Hamamatsu data

**Figure 2** Bioluminescence measurements in a human head and neck squamous carcinoma (HN 4197) in serial frozen tumour tissue sections (5 μm thickness) of (**a**) ATP, (**b**) glucose and (**c**) lactate. A native section (**d**) is included for reference.

**Figure 3** Histogram showing the distribution range between the 10th and 90th percentiles for the metabolite concentrations of (**a**) ATP, (**b**) glucose and (**c**) lactate in the tumours HN 4197, MeWo, SW 480, EO 771 described in Figures 2 and 4 6.

analysis function program described in the instruction manual and then reconverted to absolute values (concentration *vs* %) by Microsoft Excel 5. Background intensities outside the tumour image were excluded from the histograms which take only the 10th and 90th percentiles into consideration. Average metabolite values in the cryosections were calculated from the histogram distribution curves.

For comparison, average metabolite levels were also determined by measuring the total intensities of the same tumour sections, the outlines of which were previously marked by the 'free-pen window' method in the data analysis program, i.e. excluding the background light intensity frequency at the outset. The histograms were obtained and average metabolite values calculated as described above.

### Results

### *Quantification and optimisation of metabolite determination*

The bioluminescence intensity of the substrates was calibrated in absolute values using heat inactivated pig or mouse liver homogenates supplemented with different substrate concentrations previously measured by a standard enzymatic method (Bergmeyer, 1970; Tamulevicius *et al.*, 1987). This was done to achieve a similar 'tissue' situation to that in the tumour although aqueous standards produced quantitatively similar results. A linear correlation between bioluminescence intensity and metabolite concentration was seen over a physiological range for all three substrates. However, in the case of glucose, the activity of glucose-6-phosphate dehydrogenase in the enzyme cocktail needed to be increased by 60% compared with that of the original method in order to obtain a linear correlation over a concentration range of 5 mM. Under our conditions, the original method of Müller-Klieser and Walenta (1993) showed a linearity only up to 2.5 mM followed by a plateau-like level at 5 mM. Representative calibration curves for the metabolites are shown in Figure 1. Preliminary studies showed that omission of the components, glycerol, gelatin and polyvinylpyrrolidone from the various cocktails resulted in easier handling because of the lowered viscosity and better controllable consistency, while not significantly inducing smearing or smudging of the metabolite image during the corresponding integration time. However, only prolonged exposure times of approximately 5–10 min resulted in excessive diffusion of metabolites within and from the tumour.

Temporal intensity studies were carried out to determine the time course of photon yield at various concentrations of ATP, glucose and lactate. In the case of ATP, luminescence was nearly maximal after thawing to 10°C and remained relatively constant at this level for 60 s before approaching zero intensity after 2 min; an integration time of 60 s was used for the quantitative determination. Similarly, glucose and lactate reached peak intensity after an integration time of about 60–90 s after thawing, with a steady decline subsequently towards zero intensity after 5 min. For the serial determination of these substrates in both standards and cryosections, an integration time of 90 s was routinely used for measurements. Self-absorption of bioluminescence by the cryosections themselves was found to be negligible since a doubling of slice thickness resulted in a linear 2-fold increase in photon yield with either heat-inactivated liver homogenates containing different amounts of metabolites or tumour tissue itself (data not shown).

Bioluminescence data were obtained from both peripheral and central areas of the tumour with regions of interest depicting both 'hot' and 'cold' spots of metabolites; an attempt was also made to obtain an overall 'average' value of the metabolite concentration in the section based on the histogram and free-pen method for comparison with results from conventional methods of metabolite determination.

### *Determination of metabolites in tumours*

*Head and neck squamous carcinoma HN 4197* The peripheral and central areas in the head and neck carcinoma 4197 show a high degree of heterogeneity with respect to the distribution of ATP (Figure 2), the pattern of which is maintained in serial sections. Interestingly, there are 'hot-spots' with ATP levels of up to about 2 $\mu$mol g$^{-1}$ in the periphery directly adjacent to areas at the lower level of detection of approximately 0.2 $\mu$mol g$^{-1}$, although in contrast to other tumours (see below) this metabolite is also to be found in the partially necrotic regions in the centre. This tumour shows a relatively low mean ATP level of about 0.7 $\mu$mol g$^{-1}$ using the free-pen method, in close agreement with that obtained biochemically (approximately 0.9 $\mu$mol g$^{-1}$) (Table I) and from the ATP histogram with an arithmetic average of about 0.6 $\mu$mol g$^{-1}$ (Figure 3).

By contrast, glucose was found to be much more uniformly distributed over the entire region albeit at low concentrations (approximately 0.6 $\mu$mol g$^{-1}$), typically found in this tumour by conventional biochemical assays, except for

**Table I** Metabolite levels in tumours ($\mu$mol g$^{-1}$ tissue)

| | | *Bioluminescence* | | | *Biochemical* | | |
|---|---|---|---|---|---|---|---|
| *Tumour* | | *ATP* | *Glucose* | *Lactate* | *ATP* | *Glucose* | *Lactate* |
| HN 4197 | Minimum | 0.2 | 0.2 | 0.5 | | | |
| | Maximum | 2.0 | 2.5 | 7.0 | | | |
| | Mean (FP) | 0.7 | 0.5 | 3.5 | 0.9 | 0.6 | 4.8 |
| | Mean (H) | 0.6 | 0.4 | 2.9 | | | |
| MeWo | Minimum | 0.2 | 0.2 | 0.5 | | | |
| | Maximum | 5.0 | 1.5 | 12.0 | | | |
| | Mean (FP) | 2.2 | 0.6 | 7.3 | 1.8 | 0.8 | 5.5 |
| | Mean (H) | 1.6 | 0.4 | 4.7 | | | |
| SW 480 | Minimum | 0.2 | 0.2 | 1.0 | | | |
| | Maximum | 2.5 | 1.9 | 9.0 | | | |
| | Mean (FP) | 0.5 | 0.2 | 6.0 | 0.9 | 0.4 | 5.7 |
| | Mean (H) | 0.8 | 0.3 | 4.6 | | | |
| EO 771 | Minimum | 0.2 | 0.2 | 1.0 | | | |
| | Maximum | 2.5 | 1.8 | 6.0 | | | |
| | Mean (FP) | 1.8 | 0.8 | 3.5 | 1.6 | 1.4 | 4.5 |
| | Mean (H) | 1.2 | 0.5 | 2.5 | | | |
| Heart | Minimum | 0.2 | 0.2 | 0.4 | | | |
| | Maximum | 5.0 | 1.5 | 2.5 | | | |
| | Mean (FP) | 2.5 | 0.7 | 1.2 | 3.8 | 1.2 | 0.9 |
| | Mean (H) | 2.0 | 0.5 | 1.4 | | | |

FP, free pen; H, histogram. The data for the means from the histograms are based on the values ranging between the 10th and 90th percentiles.

a few sporadic sites of higher accumulation in the periphery with glucose levels of up to about 2.5 μmol $g^{-1}$, in good agreement with an averaged free-pen section value of *c.* 0.5 μmol $g^{-1}$ and average histogram value of 0.4 μmol $g^{-1}$ (Figures 2 and 3; Table I); nevertheless, most glucose levels were estimated at the lower level of detection of bioluminescence below 0.3 μmol $g^{-1}$, as reflected in the histogram.

Lactate was generally found to be significantly higher in the central areas with maximum levels of approximately 7 μmol $g^{-1}$ (yellow) than in the periphery (0.5 μmol $g^{-1}$; blue), with an overall average section content of 3.5 and 2.9 μmol $g^{-1}$ respectively by pen and histogram method, compared with 4.8 μmol $g^{-1}$ biochemically (Table I). Although the images obtained from consecutive sections showed a high degree of heterogeneity (Figure 2), both cryosections were found to have similar distribution levels in the histograms (Figure 3).

*Melanoma MeWo* The representative distribution pattern of ATP in the corresponding sequential histological sections of a human melanoma xenograft is shown in Figure 4. These show ATP to be located predominantly in the periphery with maximal concentrations of almost up to 5 μmol $g^{-1}$ clearly demarcated from areas with levels of less than 0.2 μmol $g^{-1}$ in the central region, representing the lower level of detection. The mean level of ATP in the cryosections was found to be 2.2 μmol $g^{-1}$ by the free-pen method, in good agreement

**Figure 4** Bioluminescence measurements in human melanoma xenograft (MeWo) in serial frozen tumour tissue sections (5 μm thickness), of (**a**) ATP, (**b**) glucose and (**c**) lactate.

with the biochemical determination of 1.8 μmol g$^{-1}$ (Table I), while that obtained from the histograms was 1.6 μmol g$^{-1}$. In the particular histogram shown, the mean level corresponded to approximately 1.2 μmol g$^{-1}$, reflecting the large degree of intersectional heterogeneity in ATP distribution, although all histograms were qualitatively similar in shape (Figure 3). As in the case of the squamous carcinoma HN 4197, the levels of both glucose and lactate seem to be more confined to the central region, with maximal concentrations of up to 1.5 and 12 μmol g$^{-1}$ respectively (Figure 4, Table I). The mean levels of glucose calculated by the free-pen and histogram methods were approximately 0.6 and 0.4 μmol g$^{-1}$ respectively, compared with the biochemical level of 0.8 μmol g$^{-1}$. In the case of lactate, the mean histogram level of 4.7 μmol g$^{-1}$ was found to show a better correlation with the biochemically determined level (5.5 μmol g$^{-1}$) than with that resulting from the free-pen method (7.3 μmol g$^{-1}$) (Figure 3).

*Rectum carcinoma SW 480* As shown in Figure 5, consecutive cryosections of this tumour clearly show ATP to be almost exclusively confined to the narrow rim of viable cells in the lower periphery with maximum levels of about 2.5 μmol g$^{-1}$, while the remainder of the tissue is practically devoid of this metabolite. Overall, the tumour shows a mean ATP level of 0.8 μmol g$^{-1}$ as calculated from the histogram (Figure 3) which is in good agreement with the biochemically determined value of 0.9 μmol g$^{-1}$ and slightly higher than that obtained by the pen method of 0.5 μmol g$^{-1}$ (Table I).

Glucose was found to be distributed in the tumour section in a manner similar to that seen in the squamous carcinoma with an accumulation of 'hotspots' in the peripheral region close to ATP, reaching a maximum value of about 1.9 μmol g$^{-1}$ but with a generally low overall biochemical level of 0.4 μmol g$^{-1}$, agreeing well with those obtained from the histogram and by the pen method of 0.3 and 0.2 μmol g$^{-1}$ respectively.

However, the localisation of lactate with levels of up to 9 μmol g$^{-1}$ is almost completely restricted to the vast central necrotic region almost completely lacking ATP or glucose (Figure 5), while levels of less than 1 μmol g$^{-1}$ are observed in the tumour periphery. Similarly, the mean lactate levels obtained by the different procedures (pen, histogram, biochemical) with values of 6.0, 4.6 and 5.7 μmol g$^{-1}$ respectively were also found to show good agreement (Table I; Figure 3).

*Adenocarcinoma EO771* The distribution of ATP, glucose and lactate in sections of the murine adenocarcinoma is shown in Figure 6. Here, glucose is localised almost exclusively in the upper part of the tumour with a maximum level of 1.8 μmol g$^{-1}$, while the remaining tissue contains glucose at a fairly constant level of only about 0.2 μmol g$^{-1}$ at the lower level of detection. However, the mean values of the overall tumour glucose levels of 0.8 and 0.5 μmol g$^{-1}$ calculated by pen and histogram method show a poorer correlation to the biochemically determined value of 1.4 μmol g$^{-1}$ in contrast to that seen in the above mentioned tumours (Table 1; Figure 3). It is particularly interesting that the glucose-enriched region contains almost no ATP at all, except for a distinct slight amount (approximately 0.4 μmol g$^{-1}$) in the outermost upper left tip, whereas this metabolite is predominantly located in the glucose-poor region with concentrations up to 2.5 μmol g$^{-1}$. In contrast to glucose, there is good agreement between the mean ATP levels of 1.8, 1.2 and 1.6 μmol g$^{-1}$ obtained from the pen, 'histogram and biochemical assay respectively. However, lactate appears to

**Figure 5** Bioluminescence measurements in a human colorectal adenocarcinoma (SW 480) in serial frozen tumour tissue sections (5 μm thickness), of (**a**) ATP, (**b**) glucose and (**c**) lactate. A native section (**d**) is included for reference.

be rather more uniformly and selectively distributed over the glucose-poor than glucose-rich central region with a maximum peak level of approximately 6 $\mu$mol g$^{-1}$ tissue, with levels approaching approximately 1 $\mu$mol g$^{-1}$ and less in the outer periphery. As for glucose, there is good agreement between the mean lactate levels of 3.5, 2.5 and 4.5 $\mu$mol g$^{-1}$ respectively obtained from the pen, histogram and biochemical determination (Table I; Figure 3).

*Normal mouse heart* In contrast to the situation in tumours, the three substrates ATP, glucose and lactate are distributed much more homogeneously in normal mouse heart and maintained throughout in serial cryosections (Figure 7). Here, the ventricles are clearly discernible as gaps in the luminescence images, particularly so in the case of ATP and lactate. The mean ATP content of the tissue measured biochemically was about 3.8 $\mu$mol g$^{-1}$ whereas the corresponding levels in the cryosections determined from the histogram and by the free-pen method were 2.0 and 2.5 $\mu$mol g$^{-1}$ respectively, although 'hotspots' of up to 5 $\mu$mol g$^{-1}$ were seen in the periphery (Table I). Similarly, the level of glucose in the sections as estimated by the pen and histogram method, although in good agreement with each other with values of 0.7 and 0.5 $\mu$mol g$^{-1}$ respectively, was below that determined biochemically (1.2 $\mu$mol g$^{-1}$). However, there tended to be more glucose located in the regions around the ventricles, reaching peak levels of up to about 1.5 $\mu$mol g$^{-1}$. Lactate levels of up to a maximum of 2.5 $\mu$mol g$^{-1}$ tissue were seen in the cryosections, which resulted in mean tumour levels of 1.2 and 1.4 $\mu$mol g$^{-1}$ by pen and histogram method, compared with the biochemical estimation of 0.9 $\mu$mol g$^{-1}$.

### Discussion

Compared with conventional biochemical assays resulting in a single 'global' measurement of metabolite levels from different tissues, the use of photon imaging enables these to be measured both qualitatively and quantitatively in absolute units with a high spatial resolution in particular regions of interest in the tissue. This technique is based on well-defined enzymatic reactions used in conventional biochemical analysis, but which are coupled to the use of luciferases allowing metabolites to be detected as a result of the production of bioluminescence, and has been applied to metabolic imaging in experimental animal and human solid tumours, normal tissues and multicellular spheroids (Müller-Klieser *et al.*, 1988; Walenta *et al.*, 1990; Müller-Klieser and Walenta, 1993).

At present, this method has been restricted to the determination of ATP, glucose and lactate although in theory all substrates are capable of detection provided they can be coupled to the FMN redox system via NAD(P)H and an appropriate luciferase enzyme, thus underlining its vast potential. Especially in the case of tumours, this method has the distinct advantage of revealing the often unique and characteristic heterogeneous distribution of metabolites, correlating this with the histological structure of the tissue. For example, differences in ATP levels may better reflect the spatial distribution of viable and necrotic regions of the tumour than can be demonstrated by other means. Such correlations have been shown previously in studies with multicellular spheroids where the ATP content was low in the hypoxic central necrotic core but considerably higher in the surrounding outer rim of viable oxic cells (Müller-Klieser *et*

**Figure 6** Bioluminescence measurements in a murine mammary adenocarcinoma (EO 771) in serial frozen tumour tissue sections (5 $\mu$m thickness), of (**a**) ATP, (**b**) glucose and (**c**) lactate. A native section (**d**) is included for reference.

*al.*, 1988; Walenta *et al.*, 1990), suggesting that cell death may arise as a result of a lack of energy. However, the correlation between metabolic and histological parameters in individual tumours and normal tissues would appear to be completely different and much more complex. One particularly pronounced obvious difference is the large degree of heterogeneity in the tumours studied here compared with the rather more homogeneous patterns observed in normal heart tissue. Further, it should be emphasised that these are preliminary results with respect to the number of tumours

**Figure 7** Bioluminescence measurements in serial frozen tissue sections from normal mouse heart (5 μm thickness), of (**a**) ATP, (**b**) glucose and (**c**) lactate. A native section (**d**) is included for reference as well as the histogram distribution pattern.

studied. Although the results presented here for each tumour entity are based on one tumour each, further studies on the head and neck carcinoma and adenocarcinoma with hyperthermia and or radiosensitisers to be reported elsewhere have underlined and further substantiated the nature of the distribution patterns observed here, i.e. the data are reproducible for these tumours, thus possibly permitting statements on intra- and interindividual heterogeneity to be made.

The studies by Konerding *et al.* (1989*a*, *b*) have clearly emphasised the chaotic and tortuous nature of the vasculature during tumour angiogenesis, a phenomenon not observed in normal tissues, and as a result of which, alterations in cellular biochemistry are to be expected. Walenta *et al.* (1992) have demonstrated the distribution of ATP in tumours to mainly reflect the efficiency of the microvasculature. The general pattern of an increase in ATP in the periphery of tumours may be related to an improved vascular supply here as shown by Tozer *et al.* (1990) for implanted tumours. However, this has not always been observed by other authors (Konerding *et al.*, 1989*a*, *b*) and may depend on such factors as the site of implantation, tumour size or tumour entity. This may also be the case in the tumours studied here, where the more slowly growing rectum SW 480 and head and neck HN 4197 squamous carcinomas show highly localised regions with ATP directly adjacent to vast areas largely devoid of this metabolite, while the more rapidly growing ones, MeWo melanoma and murine adenocarcinoma EO 771, present a more widespread distribution pattern. The results of the studies by Streffer *et al.* (1995) on the oxygen tensions, proliferation rates and glucose metabolism in these tumours would appear to corroborate the present bioluminescent findings. Thus, the tumour with the higher $pO_2$ (EO 771) also showed the highest mean levels of ATP using photon imaging whereas the tumours HN 4197 and SW 480, with considerably lower mean oxygen levels, have much lower levels of ATP and reflect the significance of bioluminescence studies for tumour metabolism. This also applies to some extent to glucose, where localisation of substrate may also be correlated with tumour development, but not, however, to lactate which shows no such specific confinements.

Similar findings have been made by Müller-Klieser and Walenta (1993) for these metabolites in a human melanoma xenograft, where ATP and glucose were confined to the outermost periphery with lactate assuming a more general distribution over the entire section, and a biopsy of a cervix carcinoma, with glucose present in considerable amounts over the whole section, while lactate and ATP were completely lacking over large areas.

Thus, the quantification of tumour heterogeneity in terms of metabolite distribution underlines the need for and usefulness of such bioluminescence studies, which may have prognostic relevance for human tumours. A further aim of the present study was directed towards the possible identification of parameter relationships between the metabolite distributions in the tumours. In general, the distribution of ATP showed a higher peripheral to central region (P>C) concentration whereas the reverse (P<C) was the case for lactate. The distribution of lactate is apparently largely determined through the blood flow and will accumulate in those regions where it cannot be transported from the tumour (Streffer, 1990). This phenomenon is readily apparent from the almost Gaussian distribution in the histogram (Figure 3). However, the situation is not so clear cut for glucose, two showing a higher P>C content and two with a P = C level. This large divergence in metabolite distribution also becomes apparent when considering their percentage frequency histograms. Whereas ATP is almost normally distributed in heart, none of the tumours studied show such a clear effect except perhaps for the melanoma, and no such phenomenon is seen for glucose.

The large accumulation of lactate in central areas would more closely reflect the situation with respect to glucose consumption and oxygen availability in the tumour (Kallinowski *et al.*, 1988; Streffer, 1990). An insufficient blood supply would result in hypoxic conditions leading to an enhanced conversion of glucose to lactate via glycolysis (Streffer, 1994). Although studies by Müller-Klieser *et al.* (1991) have shown a positive correlation between ATP content and oxygen supply in rodent tumours, this does not necessarily imply that there is a positive correlation between ATP and radiosensitivity or hypoxic fraction of tumours, as shown by Rofstad *et al.* (1988). Bioluminescence may be of practical clinical importance in tumour diagnosis and as an additional prognostic factor for the therapeutic outcome, since biopsies are routinely taken from patients, permitting the determination of cell death occurring after therapy.

### References

BERGMEYER H. (1970). *Methoden der Enzymatischen Analyse*. Verlag Chemie: Weinheim.

HOSSMANN K-A, MIES G, PASCHEN W, SZABO L, DOLAN E AND WECHSLER W. (1986). Regional metabolism of experimental brain tumors. *Acta Neuropathol.*, **69**, 139–147.

KALLINOWSKI F, VAUPEL P, RUNKEL S, BERG G, FORTMEYER HP, BAESSLER KH, WAGNER K, MÜLLER-KLIESER W AND WALENTA S. (1988). Glucose uptake, lactate release, ketone body turn-over, metabolic micromilieu, and pH distributions in human breast cancer xenografts in nude mice. *Cancer Res.*, **48**, 7264–7272.

KONERDING MA, STEINBERG F AND STREFFER C. (1989*a*). The vasculature of xenotransplanted human melanomas and sarcomas on nude mice. I. Vascular corrosion casting studies. *Acta Anat.*, **136**, 21–26.

KONERDING MA, STEINBERG F AND STREFFER C. (1989*b*). The vasculature of xenotransplanted human melanomas and sarcomas on nude mice. II. Scanning and transmission electron microscopic studies. *Acta Anat.*, **136**, 27–33.

LEIBOVITZ A, STINSON JC AND McCOMBS WB. (1976). Classification of human colorectal adenocarcinoma cell lines. *Cancer Res.*, **36**, 4562–4569.

MÜLLER-KLIESER W AND WALENTA S. (1993). Geographical mapping of metabolites in biological tissue with quantitative bioluminescence and single photon imaging. *Histochem. J.*, **25**, 407–420.

MÜLLER-KLIESER W, WALENTA S, PASCHEN W, KALLINOWSKI F AND VAUPEL P. (1988). Metabolic imaging in microregions of tumors and normal tissues with bioluminescence and photon counting. *J. Natl Cancer Inst.*, **80**, 842–848.

MÜLLER-KLIESER W, KRÖGER M, WALENTA S AND ROFSTAD EK. (1991). Comparative imaging of structure and metabolites in tumours. *Int. J. Radiat. Biol.*, **60**, 147–159.

PASCHEN W. (1985). Regional quantitative determination of lactate in brain sections: A bioluminescent approach. *J. Cereb. Blood Flow Metabol.*, **5**, 609–612.

PASCHEN W. (1990). Imaging of energy metabolites (ATP, glucose and lactate) in tissue sections: a bioluminescent technique. *Prog. Histochem. Cytochem.*, **20**, 1–122.

PASCHEN W, NIEBUHR I AND HOSSMANN KA. (1981). A bioluminescence method for the demonstration of regional glucose distribution in brain slices. *J. Neurochem.*, **36**, 513–517.

ROFSTAD EK, HOWELL RL, DEMUTH DP, CECKLER TL AND SUTHERLAND RM. (1988). $^{31}$P NMR spectroscopy *in vivo* of two murine tumour lines with widely different fractions of radiobiologically hypoxic cells. *Int. J. Radiat. Biol.*, **54**, 635–649.

STEINBERG F, KONERDING MA AND STREFFER C. (1990). The vascular architecture of human xenotransplanted tumors: histological, morphometrical and ultrastructural studies. *J. Cancer Res. Clin. Oncol.*, **116**, 517–524.

STREFFER C. (1990). Biological basis of thermotherapy. In *Biological Basis of Oncologic Thermotherapy*, Gautherie M. (ed.) pp. 4–71. Springer: Berlin.

STREFFER C. (1994). Glucose, energy-metabolism and cell proliferation in tumors. In *Oxygen Transport to Tissue XV*, Vaupel P, Zander R and Bruley DF. (eds) pp. 327–333. Plenum Press: New York.

STREFFER C, STEINBERG F AND TAMULEVICIUS P. (1995). Oxygenation and energy metabolism in tumors: are they correlated? In *Tumor Oxygenation*, Vaupel PW, Kelleher DK and Günderoth M. (eds) pp. 195–204. G Fischer: Stuttgart.

TAMULEVICIUS P, LUSCHER G AND STREFFER C. (1987). Effects on intermediary metabolism in mouse tissues by Ro-03-8799. *Br. J. Cancer*, **56**, 315–320.

TAMULEVICIUS P, STEINBERG F AND STREFFER C. (1992). Effect of tumor necrosis factor on tumor energy metabolism and vascularization in two different xenotransplanted tumor cell lines. In *Immunodeficient Mice in Oncology: Contributions to Oncology*, Vol 42, Fiebig HH and Berger DP (eds) pp. 272–276. Karger: Basle.

TOZER GM, LEWIS S, MICHALOWSKI A AND ABER V. (1990). The relationship between regional variations in blood flow and histology in a transplanted rat fibrosarcoma. *Br. J. Cancer*, **61**, 250–257.

WALENTA S, DOETSCH J AND MÜLLER-KLIESER W. (1990). ATP concentrations in multicellular spheroids assessed by single photon imaging and quantitative bioluminescence. *Eur. J. Cell Biol.*, **52**, 389–393.

WALENTA S, DELLIAN M, GOETZ AE, KUHNLE GE AND MÜLLER-KLIESER W. (1992). Pixel-to-pixel correlation between images of absolute ATP concentrations and blood flow in tumours. *Br. J. Cancer*, **66**, 1099–1102.